# What do people believe to be the cause of low back pain? A scoping review

**DOI:** 10.1016/j.bjpt.2023.100562

**Published:** 2023-11-07

**Authors:** Søren Grøn, Kasper Bülow, Tobias Daniel Jonsson, Jakob Degn, Alice Kongsted

**Affiliations:** a Department of Sports Science and Clinical Biomechanics, University of Southern Denmark, Campusvej 55, 5230 Odense M, Denmark; b Chiropractic Knowledge Hub, Campusvej 55, 5230 Odense M, Denmark; c Centre for Nutrition, Rehabilitation and Midwifery, University College Absalon, Slagelse, Denmark; d Independent researcher (chiropractic practice)

**Keywords:** Attitudes and beliefs, Causal beliefs, Illness perceptions, Low back pain, Questionnaire, Scoping review

## Abstract

**Objective:**

To explore how causal beliefs regarding non-specific low back pain (LBP) have been quantitatively investigated.

**Methods:**

A scoping review based on the guidelines by the JBI (former Joanna Briggs Institute) was conducted. We searched Medline, Embase, Psychinfo, and CINAHL for relevant studies and included peer-reviewed original articles that measured causal beliefs about non-specific LBP among adults and reported results separate from other belief domains.

**Results:**

A total of 81 studies were included, of which 62 (77%) had cross sectional designs, 11 (14%) were cohort studies, 3 (4%) randomized controlled trials, 4 (5%) non-randomized controlled trials, and 1 (1%) case control. Only 15 studies explicitly mentioned cause, triggers, or etiology in the study aim. We identified the use of 6 questionnaires from which a measure of causal beliefs could be obtained. The most frequently used questionnaire was the Illness Perception Questionnaire which was used in 8 of the included studies. The studies covered 308 unique causal belief items which we categorized into 15 categories, the most frequently investigated being causal beliefs related to “*structural injury or impairment”*, which was investigated in 45 (56%) of the studies. The second and third most prevalent categories were related to “*lifting and bending*“ (26 studies [32%]) and “*mental or psychological*” (24 studies [30%]).

**Conclusion:**

There is a large variation in how causal beliefs are measured and a lack of studies designed to investigate causal beliefs, and of studies determining a longitudinal association between such beliefs and patient outcomes. This scoping review identified an evidence gap and can inspire future research in this field.

## Highlights

• There is a high variation in measuring causal beliefs about low back pain.
• No measurement exists that clearly isolates causal beliefs from other belief domains.
• There is a lack of studies exploring longitudinal relationships between causal beliefs and health outcomes.
• Causal beliefs are just one element of a complex beliefs construct, and there is very little quantitative evidence from which its unique relevance can be judged.

## Introduction

The way people understand pain can influence their conscious or unconscious response to it, thus pain perceptions impact behavior and pain related disability.^1^^,^^2^ This is outlined in the Common-Sense Model which illustrates that people create cognitive representations to make sense of an experience, e.g., when experiencing pain.^2^^,^^3^ The representation of illness is created from developing a coherent understanding across the following belief domains: a) what is this pain? (Identity beliefs), b) what caused this pain? (Causal beliefs), c) what will this pain mean to me? (Consequence beliefs), d) how can I control this pain? (Control beliefs), and e) how long will it last? (Timeline beliefs).^1^^,^^2^ The theory suggests that people try to make a coherent understanding of an illness which drives actions and behaviors in response to that illness.

In low back pain (LBP), qualitative research indicates that causal beliefs can have an immense impact on people's lives and how they manage their LBP.^4^, ^5^, ^6^, ^7^ For instance, believing that LBP is caused by damage or the spine being weak can lead to overprotective behavior that involves avoiding certain movements or valued activities.^5^, ^6^, ^7^, ^8^, ^9^ Furthermore, such causal beliefs about LBP may be a barrier to modern guideline-based care for LBP, as it seems some patients feel miscast for self-management interventions because it does not match their illness beliefs.^10^

Multiple questionnaires exist regarding beliefs about LBP, and some include questions reflecting causal beliefs. For instance, the belief that LBP is caused by damage or injury of an organic structure is measured in the Pain Beliefs Questionnaire (PBQ) asking if “*Pain is the result of damage to the tissue of the body*” and as part of the Back Pain Attitudes Questionnaire (Back-PAQ): “*Back pain means you have injured your back”*.^11^^,^*^12^* Both are examples of single items in questionnaires that investigate multiple domains of beliefs. The widely used illness perception questionnaire (IPQ) also includes causal belief items.^13^ However, a systematic review from 2018 that investigated the association between IPQ scores and pain and disability among people with musculoskeletal pain did not include causal beliefs because these are not measured on a numeric scale in the IPQ.^14^

Thus, as summarized above, it seems from qualitative research that causal beliefs may be highly important in LBP and several questionnaires exist to potentially investigate this quantitatively. However, the quantitative measure of causal beliefs seems to be heterogeneous and there is currently no overview of the literature investigating causal beliefs. It is thus unclear what quantitative evidence exists that isolates the importance of causal beliefs in LBP from other belief domains. To investigate if the relationship seen in qualitative studies between causal beliefs and poor outcomes of LBP has been investigated in quantitative studies, we conducted a scoping review to map out this research. The aim was to provide an overview of how causal beliefs regarding non-specific LBP have been quantitatively investigated. The specific objectives were to examine: a) What questions and questionnaires have been used to measure causal beliefs regarding non-specific LBP? b) What types of causal beliefs about non-specific LBP have been identified and how many studies have investigated these beliefs? c) In which type of studies and contexts have causal beliefs about non-specific LBP been measured? and d) What outcomes have been investigated for an association with causal beliefs about non-specific LBP in cross-sectional and longitudinal designs?

## Methods

The protocol for this scoping review was pre-registered at Open Science Framework on December 20, 2021, and is available at https://osf.io/7hezb. The method was based on the instructions provided in the JBI manual of evidence synthesis on scoping reviews, and we reported the review according to the PRISMA-ScR checklist for scoping reviews.^15^^,^^16^

### Eligibility criteria

We included published original scientific papers that measured causal beliefs about non-specific LBP and reported results from this domain that could be isolated from other beliefs domains. Population were adults from non-clinical populations with or without non-specific LBP, health-care providers and clinical (i.e., care seeking) populations of patients with non-specific LBP*.* We excluded studies testing psychometric properties of questionnaires or transcultural adaptations.

Our interpretation of causal beliefs was conceptualized by the common-sense model, which implies that the perception of what caused LBP should be distinguishable from beliefs that according to the common-sense-model relate to other domains. We defined causal beliefs as: a) a perceived cause of LBP, b) a perceived trigger of a new onset of LBP, or c) a perceived risk factor for LBP. Any quantitative measure or data from quantified text responses capturing a causal belief was included. Thus, studies measuring causal beliefs by text responses were only included if the researchers categorized and quantified the text responses in the studies. Studies that in the abstract mentioned causal beliefs specifically or unspecified beliefs were included for full text assessment. Studies measuring beliefs that were specified as other types of beliefs than causal beliefs (e.g., fear avoidance beliefs or kinesiophobia beliefs) were not included.

Furthermore, only peer-reviewed articles written in English were included. We did not use any restriction on time period.

### Search strategy

We searched the following electronic databases: Embase, Medline, PsychInfo, and CINAHL. The search strategy was developed in collaboration with a librarian from University Library of Southern Denmark and was initially developed for Embase and then adapted to the other databases. Keywords and search terms were identified from preliminary searches and reading of articles related to the subject. The search combined words of LBP with words for causal beliefs, using both keywords and subject headings (Supplementary material A). The search was conducted on January 10, 2022.

### Selection of sources of evidence

Duplicates were removed in Endnote before uploading citations to Covidence review software (Veritas Health Innovation, Melbourne, Australia) for screening and data extraction. Prior to screening of titles and abstracts the inclusion criteria were tested by the entire review team in a pilot screening of a small test sample of 30 titles and abstracts. Two rounds of pilot screening were completed to achieve the desired 75% agreement threshold.

The screening of titles and abstracts as well as full-text assessments were done double-blinded with SG screening the entire sample and TJ, KB, and JD splitting the sample between them. Disagreements between the reviewers were settled through discussion to reach consensus.

### Data charting process

The extracted data included study characteristics (population, setting, country, and aim), causal beliefs measurement tool, causal belief items, and outcomes investigated for cross-sectional or longitudinal associations with causal beliefs. The outcomes investigated were only extracted in cases where the association could be linked to the isolated causal belief item. SG and AK piloted the extraction tool and made modification before moving on to the final extraction. Extraction was done independently by SG extracting the entire sample of studies and AK, TJ, JD, and KB splitting the sample between them.

Prior to data extraction consensus between the review team was made to determine which items from the identified questionnaires were considered causal beliefs (Supplementary material B). Each member independently voted for each item whether they deemed it to measure a causal belief based on face validity. The votes were compared, and disagreements settled by discussions in the entire review team to reach consensus.

### Synthesis of results

Data were exported from Covidence and handled in Stata/MP V.17. (StataCorp Texas, USA). Extracted data were organized in tables and visualized in bar charts made in Microsoft Excel (Microsoft Corporation, Redmond, WA). The causal belief items extracted from the studies were categorized into mutually exclusive categories based upon face validity of the beliefs. The first half of the items were categorized in a consensus forum between SG, JD, KB, and AK. The remaining were categorized by SG whereupon all authors commented and agreed upon the final categorization (for resulting categories see supplementary material C).

## Results

**Fig. 1:**
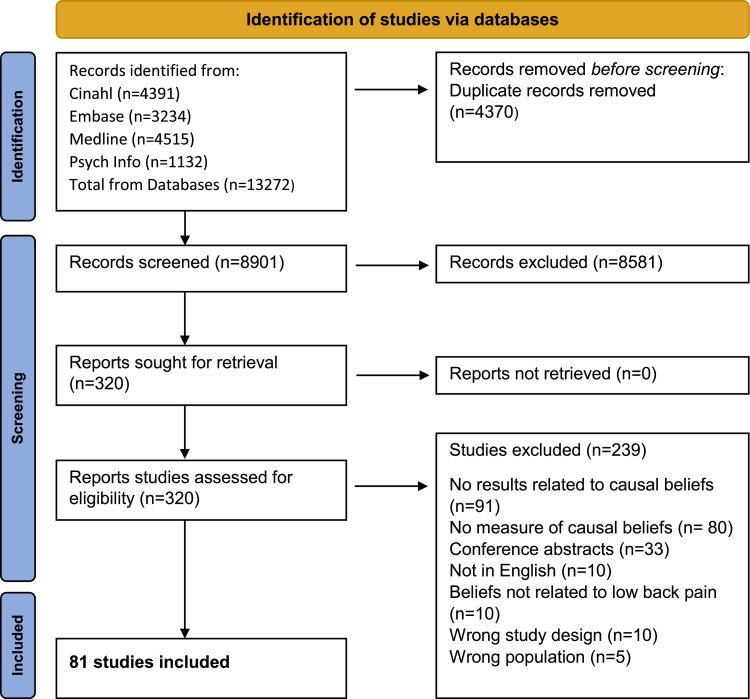
Flowchart of selection process.

Of 8901 titles and abstracts screened, 316 were assessed in full-text, and 81 papers were included (Fig. 1). Most exclusions after full text assessment were because the studies did not measure causal beliefs or did not report a result specifically related to the causal belief. Most of the studies (*n* = 62 [77%]) had cross sectional designs, 11 (14%) studies were cohorts, 3 (4%) randomized controlled trials, 4 (5%) non-randomized controlled trials, and 1 (1%) was a case-control study (Table 1). Beliefs, attitudes, opinions, myths, or perceptions were mentioned in the aim in 44 (54%) studies, and 15 (19%) had an aim specifically mentioning cause, triggers, or etiology. Thirty-three (41%) of the study samples were from the general population, 26 (32%) from health care providers, 13 (16%) from clinical population, 5 (6%) from health-care students, and 4 (5%) studies included mixed populations (Table 1). Most studies were from Western Europe, Australia, or North America.

**Table 1:** Characteristics of included studies.

Study	Country	Population	Design	Categories of causal belief measured	Measurement	Associations investigated
Lindström 1994^39^	Sweden	General population	Cohort study	LB, LMPC, PWS, PP, MP, EE	Other non-validated	No
Christe 2021^40^	Switzerland	General population	Cross-sectional study	SIP	Back-PAQ-34	No
Pereira 2020^17^	Portugal	Clinical	Cross-sectional study		IPQ-revised	Cross-sectional: pain intensity, disability, IPQ-domains, suffering, psychology morbidity
Zusman 1984^41^	Australia	Clinical	Cross-sectional study	SIP	Other non-validated	no
Talbott 2009^42^	United States	General population	Cross-sectional study	LB	Other non-validated	no
Dean 2011^43^	New Zealand	General population	Cross-sectional study	LB, PAS, LMPC, PWD, PP, TM, Gen, GHL	IPQ-brief	no
Matsui 1997^44^	Japan	General population	Cross-sectional study	LB, PAS, PP, TM, Unk	Other non-validated	Cross-sectional: physical work demand
Byrns 2004^34^	United States	Health-care providers	Cross-sectional study	PWD, OWD, Spi, Oth	Modification of Worker attributions scale, and additional questions	no
Ree 2016^45^	Norway	General population	Randomised controlled trial	LB	Deyo's back pain myths	Cross-sectional: days of sick leave
Moffett 2000^20^	UK	General population	Cross-sectional study	SIP, GHL	Other non-validated	Cross-sectional: no back pain, back pain within the past year, consulted General Practitioner for back pain within the last year
Keeley 2008^46^	UK	Clinical	Cohort study	PWD, TM, Unk	Other non-validated	Longitudinal: health related quality of life, number of health care contacts
Vargas-Prada 2012^29^	Spain	Mixed	Cohort study	OWD	Questions adapted from FABQ	Longitudinal: new LBP, new disabling LBP, persistence of LBP, persistence of disabling LBP
Scholey 1989^47^	UK	Mixed	Cross-sectional study	LB, TM	Other non-validated	no
Adhikari 2014^48^	Nepal	Health-care providers	Cross-sectional study	LB, PWD, OWD, PP	Other non-validated	no
Battista 2021^49^	Italy	General population	Cross-sectional study	PAS	Other non-validated	no
French 1997^50^	Hong Kong	Health-care providers	Cross-sectional study	LMPC, PWD, PP	Other non-validated	no
Sadeghian 2013^30^	Iran	Mixed	Cohort study	OWD	Other non-validated	Longitudinal: reporting LBP
Alshehri 2020^51^	Saudi Arabia	Health-care providers	Cross-sectional study	SIP, MP, Unk,	PABS-PT (19-items)	no
Christe 2021^40^	Switzerland	Health-care providers	Cross-sectional study	SIP	Back-PAQ-34	Cross-sectional: Degree of evidence-concordant clinical decisions for young woman with acute LBP and no sign of serious pathology
Ross 2014^52^	United States	Health-care providers	Cross-sectional study	SIP	Other non-validated	no
Werner 2007^53^	Norway	General population	Cohort study	SIP	Deyo's back pain myths	no
Stevens 2016^54^	Australia	Mixed	Cross-sectional study	LB, PAS, LMPC, PP, SIP, TM, MP, GHL, Oth	Other non-validated	no
Fitzgerald 2020^55^	Australia	Health-care providers	Cross-sectional study	SIP, MP, Unk	PABS-PT-19, ABS-MP, NPQ	no
Mehok 2019^56^	United States	General population	Cross-sectional study		Other non-validated	Cross sectional: body weight treatment recommendations
Benny 2020^57^	Canada	Health-care providers	Cross-sectional study	SIP, MP, Unk	PABS-PT (19-items)	no
Ihlebæk 2004^22^	Norway	Health-care providers	Cross-sectional study	LB, SIP	Deyo's back pain myths	Cross-sectional: sex, age, profession
Ihlebæk 2005^58^	Norway	General population	Cross-sectional study	LB, SIP	Deyo's back pain myths	no
Adams 2013^59^	United States	Health-care providers	Cross-sectional study	PWD, SIP	Modification of the standardized Nordic Questionnaire	no
Boschman 2012^60^	The Netherlands	General population	Cohort study	OWD	Other non-validated	no
James 2018^61^	Australia	General population	Cross-sectional study	OWD	Other non-validated	no
Cherkin 1988^62^	United States	Health-care providers	Cross-sectional study	SIP, MP, Unk	Other non-validated	no
Brennan 2007^63^	Ireland	General population	Cross-sectional study	LB, PAS, TM	Other non-validated	no
Goubert 2003^21^	Belgium	General population	Cross-sectional study	SIP	Low back pain beliefs questionnaire, specifically developed based on Deyo's myths, TSK, PABS-PT, and the self-care orientation scale	Cross-sectional: pain grade
Werner 2008^64^	Norway	General population	Cohort study	SIP	Deyo's back pain myths	Cross-sectional and longitudinal: Odds ratios for appropriate responses in intervention vs control counties.
Walker 2004^27^	Australia	General population	Cross-sectional study	PAS, OWD, PP, TM	Other non-validated	Cross-sectional: Logistic regression assessing the odds-ratio for care seeking using all other categories as a reference group.
Vujcic 2018^23^	Serbia	Health-care students	Cross-sectional study	PAS, PP, MP, EE, Oth	Other non-validated	Cross-sectional: sex
Maselli 2021^65^	Italy	General population	Cross-sectional study	PAS	Other non-validated	no
Patel 2016^24^	Canada	Health-care providers	Cross-sectional study	SIP	Other non-validated	Cross-sectional: sex, years of practice, hours of practice/week, population size of practice
Tarimo 2017^66^	Malawi	Clinical	Cross-sectional study	LB, PAS, OWD, PP, SIP, TM, MP, GHL, EE, Unk, Oth	Modification of LBP knowledge questionnaire	no
Dabbous 2020^67^	Lebanon	Health-care providers	Cross-sectional study	SIP	Other non-validated	no
Ross 2018^68^	United States	Health-care providers	Cross-sectional study	SIP	Other non-validated	no
Lobo 2013^69^	India	General population	Cross-sectional study	PAS, Gen, GHL	Other non-validated	no
Buchbinder 2007^70^	Australia	Health-care providers	Cross-sectional study	SIP	Other non-validated	no
Ulaska 2001^71^	Finland	General population	Case control study	LB, PAS, PP, EE	Other non-validated	no
Foster 2008^31^	UK	Clinical	Cohort study	MP, GHL, Unk, Oth	IPQ-revised	Longitudinal: disability, global rating
Glattacker 2012^32^	Germany	Clinical	Non-randomised experimental study	Gen, MP, Unk, Oth	IPQ-revised	no
Li 2020^72^	China	General population	Cross-sectional study	PAS, SIP, GHL, Spi	Other non-validated	no
Roussel 2016^73^	Belgium	Clinical	Cross-sectional study	PAS, LMPC, OWD, PP, SIP, TM, Gen, MP, GHL, EE, Unk, Oth	IPQ-revised	no
Werner 2008^74^	Norway	Health-care providers	Non-randomised experimental study	SIP	Deyo's back pain myths	Longitudinal: work in campaign area or in control area
Houben 2005^75^	The Netherlands	Health-care providers	Cross-sectional study	SIP, MP, Unk	PABS-PT (31 items)	no
Ostelo 2003^76^	The Netherlands	Health-care providers	Cross-sectional study	LMPC, SIP, MP, Unk	PABS-PT in its development form	no
Lefevre-Colau 2009^77^	France	Clinical	Cross-sectional study	OWD, PP, TM,	Other non-validated	no
Osborne 2013^78^	Ireland	General population	Cross-sectional study	LB, LMPC, PWD, TM, Unk	Other non-validated	no
Igumbor 2003^79^	Zimbabwe	Health-care providers	Cross-sectional study	LB, LMPC, PWD, OWD	Other non-validated	no
Shaheed 2015^80^	Australia	Health-care providers	Non-randomised experimental study	SIP	Pharmacists Back Beliefs Questionnaire	no
Shaheed 2017^81^	Australia	Health-care students	Non-randomised experimental study	SIP	Modified Back beliefs questionnaire	no
Johnsen 2018^82^	Norway	General population	Randomised controlled trial	LB, SIP	Deyo's back pain myths	no
Odeen 2013^83^	Norway	General population	Randomised controlled trial	LB	Deyo's back pain myths	no
Buchbinder 2009^84^	Australia	Health-care providers	Cross-sectional study	SIP	Other non-validated	Cross-sectional: special interest in LBP
McCabe 2019^85^	Ireland	Health-care students	Cross-sectional study	LB, SIP	Deyo's back pain myths	Cross-sectional: LBP teaching in medical school
Wilgen 2013^86^	The Netherlands	General population	Cross-sectional study	LB, PAS, OWD, PP, SIP, TM, Gen, MP, GHL, EE	IPQ-revised; Other: converted to IPQ R back pain	no
Munigangaiah 2016^25^	Ireland	General population	Cross-sectional study	LB, SIP	Deyo's back pain myths	Cross-sectional: sex, education, age
Coggon 2012^87^	18 different countries: Brazil, Ecuador, Colombia, Costa Rica, Nicaragua, UK, Spain, Italy, Greece, Estonia, Lebanon, Iran, Pakistan, Sri Lanka, Japan, South Africa, Australia, New Zealand	General population	Cohort study	OWD	Other non-validated	no
Darlow 2014^88^	New Zealand	General population	Cross-sectional study	SIP	Back-PAQ-34	no
Campbell 2013^18^	UK	Clinical	Cross-sectional study	MP, GHL, Unk, Oth	IPQ-revised	Cross-sectional: pain, disability
Steffens 2014^89^	Australia	Health-care providers	Cross-sectional study	LB, PAS, LMPC, PP, SIP, TM, Gen, MP, GHL	Other non-validated	no
Kent 2005^90^	Australia	Health-care providers	Cross-sectional study	PP, SIP		no
Wolter 2011^91^	Germany	Clinical	Cross-sectional study	LMPC, TM, Gen, MP, GHL, Unk, Oth	Based on the German Pain Questionnaire	no
Christe 2021^40^	Switzerland	Health-care students	Cohort study	SIP	Back-PAQ-34	no
Campbell 2004^92^	UK	Clinical	Cross-sectional study	LB, PAS, PP, SIP, TM, Gen, MP, GHL, Oth	Other non-validated	no
SilvaParreira 2015^37^	Australia	Clinical	Cross-sectional study	LB, PAS, LMPC, PP, TM, GHL, EE, Oth	Other non-validated	Cross-sectional: developing acute LBP
Igwesi-Chidobe 2017^26^	Nigeria	Clinical	Cross-sectional study	SIP, Gen, GHL, Spi	IPQ-brief	Cross-sectional: disability
Pierobon 2020^28^	Argentina	General population	Cross-sectional study	SIP	Back-PAQ-34	Cross-sectional: having seen a health care professional
Pagare 2015^93^	India	General population	Cross-sectional study	LB, SIP	Deyo's back pain myths	no
Byrns 2002^33^	United States	General population	Cross-sectional study	OWD, Spi, Oth	Other non-validated	Cross-sectional: LBP
Linton 1993^38^	Sweden	General population	Cross-sectional study	LB, PWD, OWD, PP, MP	Other non-validated	Cross-sectional: job type, upper management, lower management, blue collar
Pincus 2007^94^	UK	Health-care providers	Cross-sectional study	SIP, MP	ABS-MP	no
Leysen 2020^95^	Belgium and the Netherlands	Health-care students	Cross-sectional study	SIP, MP, Unk	PABS-PT (19-items)	no
Bar-Zaccay 2018^96^	UK	Health-care providers	Cross-sectional study	SIP, MP	PABS-PT (19-items)	no
Ihlebæk 2003^97^	Norway	General population	Cross-sectional study	LB, SIP	Deyo's back pain myths	Cross sectional: living in rural/urban area, age, education
Grimshaw 2011^98^	UK	Health-care providers	Cohort study	OWD, MP, GHL, EE, Unk, Oth	Other non-validated	Cross-sectional: use of radiographs

ABS-MP, Attitudes to Back Pain Scale in Musculoskeletal Practitioners; Back-PAQ, Back Pain Attitudes Questionnaire; EE, External environment; FABQ, Fear Avoidance Belief Questionnaire; Gen, Genetic; GHL, General health and lifestyle; IPQ, Illness perception questionnaire; LB, Lifting and bending; LBP, low back pain; LMPC, Loading, movement and physical capacity; MP, Mental/psychological; NPQ, Neurophysiology of pain questionnaire; Oth, Other; OWD, Other work demands; PABS-PT, Pain Attitudes Belief Scale for Physiotherapists; PAS, Physical activity and sports; PP, Posture and position; PWD, Physical word demands; SIP, Structural injury/impairment; Spi, Spiritual; TM, Trauma mechanism; TSK, Tampa Scale of Kinesiophobia; Unk, Unknown.

### Questions and questionnaires used to measure causal beliefs regarding non-specific LBP

**Fig. 2:**
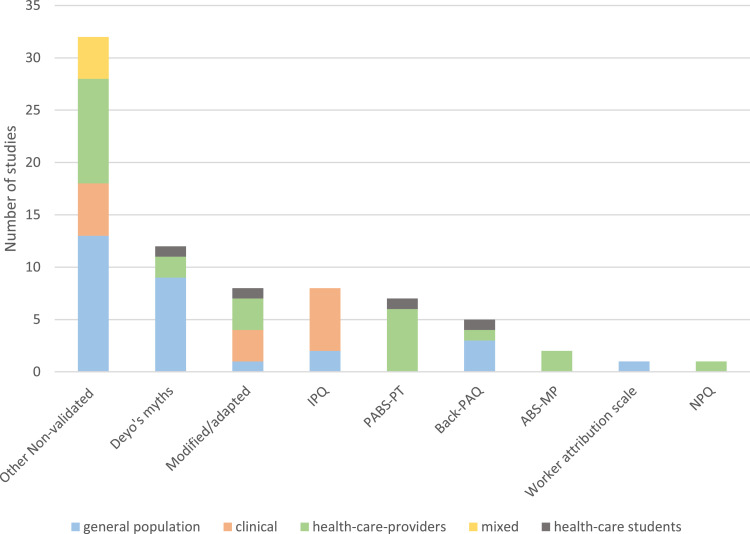
The frequency of used questions / questionnaires distributed by population. ABS-MP, Attitudes to Back Pain Scale in Musculoskeletal Practitioners; Back-PAQ, Back pain attitudes belief scale; IPQ, Illness Perception Questionnaire; NPQ, Neurophysiology of pain questionnaire; PABS-PT, Pain Attitudes and Belief Scale for Physiotherapists.

We identified the following questionnaires from which causal beliefs were obtained: Pain Attitudes and Belief Scale for Physiotherapists (PABS-PT) (7 studies) in which 7 items were deemed to be causal beliefs, Back pain attitudes belief scale (Back-PAQ) (5 studies, 2 items), Illness Perception Questionnaire (IPQ) (8 studies, 1 section), Attitudes to Back Pain Scale in Musculoskeletal Practitioners (ABS-MP) (2 studies, 1 item), Neurophysiology of pain questionnaire (NPQ) (1 study, 5 items), and the Worker Attribution Scale (WAS) (1 study, 1 section). Additionally, questions based on two of “Deyo's myths” regarding low back pain were used in 12 studies. For the remainder of the studies, eight measured causal beliefs using modification or adaptations of other questionnaires and 32 measured causal beliefs by other non-validated questionnaires or items specifically developed for the purpose of the study. Fig. 2 shows the use of the measurements within the investigated populations.

### Types of causal beliefs and number of studies investigating these

**Fig. 3:**
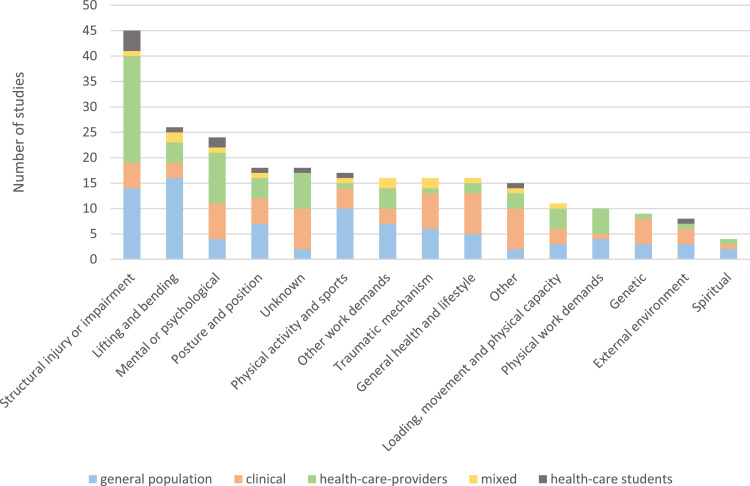
The frequency of studies investigating each category of causal beliefs distributed by population.

A total of 308 unique causal belief items were identified and categorized into 15 mutually distinct categories. All categories are explained in Table 2, and an in-depth description of items included in each category can be found in Supplemental Material C: Full list of items. The most prevalent investigated category was causal beliefs related to “*structural injury or impairment”*, which was investigated in 45 (56%) of the studies. The second and third most prevalent categories were related to “*lifting and bending*“ (26 studies [32%]) and “*mental or psychological*” (24 studies [30%]) (Fig. 3).

**Table 2:** Categories of causal beliefs.

Category	Substance
Lifting and bending	Beliefs that low back pain (LBP) is caused by lifting, bending, twisting or a combination, and also the item “*most back pain is caused by injury or heavy lifting*”.
Physical activity and sports	Beliefs that LBP is caused by exercise, sports, and other types of physical activity. This included either too much or too little exercise.
Loading, movement, and physical capacity	Beliefs that LBP is caused by repeated, specific, or sudden movements that is not explicitly related to lifting or bending, e.g., “*unexpected loads*” and “*overuse*”.
Physical work demands	Beliefs that LBP is caused by specific job tasks with a focus on the physical aspect, e.g., ”*transferring patients*” or “*physical workloads*”.
Other work demands	Beliefs that LBP is caused by non-physical (or not solely physical) work demands for instance “*heavy mental workload*” or “*a poor working environment*”.
Posture and position	Beliefs that LBP is caused by posture for instance “*poor posture*”. Also driving, sitting, and standing were included in this category.
Structural injury or impairment	Beliefs that LBP is always caused by a structural injury or that radiographs can identify the cause of LBP. Items such as “*muscle strain*” and “*disc problem*” were included in this category.
Trauma mechanism	Beliefs that LBP is caused by trauma, sport injury, or fall.
Genetic	Belief that LBP is caused by genetic factors, heredity, or related to sex.
Mental or psychological	Beliefs that LBP is caused by mental stress or other psychological factors.
General health and lifestyle	Beliefs that LBP is caused by a non-musculoskeletal health condition such as diabetes or pregnancy, or by lifestyle factors such as smoking and nutrition.
External environment	Beliefs that LBP is caused by something external, this could be weather conditions, familial problems, social factors (other than work related), shoes, or mattresses.
Spiritual	Beliefs that LBP is caused by fate, energy status, or the imbalance of the five elements.
Unknown	Beliefs that the cause of LBP is unknown or that the respondents did not know the cause of their LBP
Other	This category contained 15 items that could not be allocated to any other category such as previous LBP episodes, behavioral factors, and fatigue.

Among the frequently used questionnaires, PABS-PT contained items from the categories “*structural injury or impairment*”, “*mental or psychological*”, and “*unknown*”. Back-PAQ contained only items from “*structural injury or impairment*”. The questions based on Deyo's myth contained items from “*structural injury or impairment*”, “*lifting and bending*”, and *“unknown*”. IPQ had, due to its free text option, the capability to contain all the categories of causal beliefs created for this review.

### Outcomes investigated for an association with causal beliefs

Twenty-eight studies investigated an association between causal beliefs and other factors. Twelve studies (43%) were conducted in the general population, 6 (21%) in clinical populations, 6 (21%) among health-care providers, 2 (7%) in mixed populations, and 2 (7%) among health-care students. Cross-sectional associations were reported in 22 studies (Table 1). The most common cross-sectional associations investigated were with pain,^17^, ^18^, ^19^, ^20^, ^21^ sex,^22^, ^23^, ^24^, ^25^ disability,^17^, ^18^, ^26^ and care seeking.^20^^,^^27^^,^^28^ Longitudinal associations were investigated in 8 studies. The longitudinal association most commonly investigated was reporting LBP^29^^,^^30^ (Table 1).

## Discussion

This scoping review investigated how causal beliefs regarding non-specific LBP have been quantitatively investigated in peer reviewed scientific literature. Eighty-one studies were included accounting for 308 unique causal belief items categorized into 15 categories. Causal beliefs were most often investigated in high-income countries and most often in the general population followed by populations of health-care providers and clinical populations. The most frequent causal beliefs investigated related to structural injury or impairment, lifting and bending, and mental or psychological factors. We identified the use of 6 questionnaires from which a measure of causal beliefs could be obtained. Most of the included studies used cross-sectional designs, and 28 investigated an association between causal beliefs and other factors. Only 8 studies investigated a longitudinal relationship.

Among the questionnaires identified, only the IPQ, PBQ, and WAS were developed with the purpose of specifically measuring causal beliefs.^11^^,^^13^^,^^33^^,^^34^ However, in our review we did not find any study that reported results related to the causal belief items of the PBQ in isolation, and thus no studies using the PBQ were included. The PBQ consists of two subscales differentiating between organic and psychological beliefs, however these scales include both causal beliefs and consequence beliefs and therefore did not meet our criteria for separate information on causal beliefs.^11^ The PABS-PT and Back-PAQ were not developed to specifically measure causal beliefs.^12^^,^^35^ Yet we deemed both to have items measuring causal beliefs, and several studies using either PABS-PT or Back-PAQ were included in our review.

Although 81 studies were included, only 15 had an aim that specifically mentioned cause, triggers, or etiology. This indicates a lack of studies that are designed to investigate causal beliefs. Additionally, only 8 studies investigating longitudinal associations with causal beliefs were included in our review. In contrast, a 2019 Cochrane review of recovery expectations (Timeline beliefs) in people with LBP included 52 longitudinal studies for a narrative synthesis.^36^ Thus, it seems that expectations beliefs have been more thoroughly investigated than causal beliefs.

Causal beliefs appear to be essential for the construct of illness beliefs.^1^^,^^4^^,^^5^^,^^7^^,^^9^ However, to determine the clinical contribution of causal beliefs it is necessary that they are measured and reported in consistent ways. This would help quantify a proposed behavior reaction based on causal beliefs. The findings of this study illustrates that this can be challenging with the current existing evidence due to the large variation in the measure of causal beliefs. The variation additionally implies that causal beliefs are complex and often interacts with other types of beliefs to make up an illness representation.

### Strengths and limitations

The review followed a stringent method and was reported in accordance with current guidelines to ensure high transparency with the choices made in the process. A main concern was that it is not clear cut what constitutes a causal belief, and we consider it a strength that our definition of causal beliefs was based on the common-sense model and the question “what caused my LBP” or “what causes LBP”. However, in the review process we realized that beliefs related to triggers of back pain and contributing factors relate to this domain and thus were eligible for inclusion. For instance, the question “*what do you believe may have triggered your LBP?*”^37^ and also questions where participants rated how important they believed different items were in causing back pain, were both deemed to be a measure of causal beliefs.^38^ As these types of beliefs were discovered in the review process, specific search terms for these were not included in our search strategy. We acknowledge that relevant studies may have been missed on this account, but do not consider this a major flaw because we used a broad search strategy and screened a large number of studies.

We were strict on not including aggravating factors as causal beliefs. However, aggravating factors overlap with causal beliefs. For instance, the item from Back-PAQ “*Stress in your life (financial, work, relationship) can make back pain worse*” was deemed as measuring aggravating factors and not as a causal belief. This distinction may have favored biomedical beliefs and specific structural causes of LBP while items reflecting psychosocial causes may more often be presented as aggravating factors than as an initial cause. It can be argued that a focus on “*contributing factors”* to LBP would have been more inclusive but would also make the differentiation from other belief domains less clear. The overlap between domains made the isolation of causal beliefs challenging in some studies. Additionally, many studies had a vague description of methodology and how they measured beliefs. Thus, some subjective interpretation was inevitable.

We did not look for gray literature as we decided to limit the scoping review to peer reviewed literature. Thus, additional knowledge regarding measuring of causal beliefs may exist. However, we have no reasons to believe this would change the general findings of this review.

## Conclusion

We wanted to explore how causal beliefs regarding LBP have been quantitatively investigated and settle whether there is available evidence to quantify the impact of causal beliefs on outcomes for LBP that has been observed in qualitative studies. Based on the current evidence this is not feasible due to the large variation in measuring causal beliefs and the lack of studies designed to investigate causal beliefs and of studies determining a longitudinal association between such beliefs and patient outcomes. One belief domain does not exist in isolation from others. However, to understand unique contributions of causal beliefs it would be necessary to develop new measurement tools. This scoping review identified an evidence gap and can inspire future research in this field including search strategies and development of relevant questions and questionnaires.

## Conflicts of interest

The authors report the following potential conflict of interest: AK's position at the University of Southern Denmark is financially supported by an unrestricted grant from the Danish Foundation for Chiropractic Research and Postgraduate Education. The funders were not involved in defining the research question, designing the study, analyzing the data, or interpreting the results.

## Appendix Supplementary materials

Supplemental Material Supplemental material A: Search strategy Supplemental material B: Consensus chart for causal belief items Supplemental material C: Full list of items forming the categories of beliefs Image, application 1

## References

[1] Bunzli S., Smith A., Schütze R. (2017). Making sense of low back pain and pain-related fear. J Orthop Sports Phys Ther.

[2] Leventhal H., Diefenbach M., Leventhal E.A. (1992). Illness cognition: using common sense to understand treatment adherence and affect cognition interactions. Cognit Ther Res.

[3] Leventhal H., Phillips L.A., Burns E. (2016). The Common-Sense Model of Self-Regulation (CSM): a dynamic framework for understanding illness self-management. J Behav Med.

[4] Bonfim I.D.S., Corrêa L.A., Nogueira L.A.C. (2021). Your spine is so worn out' - the influence of clinical diagnosis on beliefs in patients with non-specific chronic low back pain - a qualitative study. Braz J Phys Ther.

[5] Darlow B., Dowell A., Baxter G.D. (2013). The enduring impact of what clinicians say to people with low back pain. Ann Fam Med.

[6] Lin I.B., O'Sullivan P.B., Coffin J.A. (2013). Disabling chronic low back pain as an iatrogenic disorder: a qualitative study in Aboriginal Australians. BMJ Open.

[7] Stenberg G., Fjellman-Wiklund A., Ahlgren C. (2014). I am afraid to make the damage worse'–fear of engaging in physical activity among patients with neck or back pain–a gender perspective. Scand J Caring Sci.

[8] Bunzli S., Smith A., Watkins R. (2015). What do people who score highly on the Tampa Scale of Kinesiophobia really believe?: a mixed methods investigation in people with chronic nonspecific low back pain. Clin J Pain.

[9] Darlow B. (2016). Beliefs about back pain: the confluence of client, clinician and community. Int J Osteopath Med.

[10] Joern L., Kongsted A., Thomassen L. (2022). Pain cognitions and impact of low back pain after participation in a self-management program: a qualitative study. Chiropr Man Therap.

[11] Edwards L.C., Pearce S.A., Turner-Stokes L. (1992). The Pain Beliefs Questionnaire: an investigation of beliefs in the causes and consequences of pain. Pain.

[12] Darlow B., Perry M., Mathieson F. (2014). The development and exploratory analysis of the Back Pain Attitudes Questionnaire (Back-PAQ). BMJ Open.

[13] Weinman J., Petrie K.J., Moss-morris R. (1996). The illness perception questionnaire: a new method for assessing the cognitive representation of illness. Psychol Health.

[14] de Raaij E.J., Ostelo R.W., Maissan F. (2018). The Association of illness perception and prognosis for pain and physical function in patients with noncancer musculoskeletal pain: a systematic literature review. J Orthop Sports Phys Ther.

[15] Micah D.J. Peters, C.G., Patricia McInerney, Zachary Munn, Andrea C. Tricco, Hanan Khalil. Scoping reviews (2020 version). JBI: Available at https://synthesismanual.jbi.global. 2020.

[16] McGowan J., Straus S., Moher D. (2020). Reporting scoping reviews-PRISMA ScR extension. J Clin Epidemiol.

[17] Pereira M., Roios E., Leite A. (2020). Subjective suffering in patients with low back pain. Int J Rheum Dis.

[18] Campbell P., Bishop A., Dunn K.M. (2013). Conceptual overlap of psychological constructs in low back pain. Pain.

[19] Glattacker M., Heyduck K., Meffert C. (2013). Illness beliefs and treatment beliefs as predictors of short-term and medium-term outcome in chronic back pain. J Rehabil Med.

[20] Moffett J.A.K., Newbronner E., Waddell G. (2000). Public perceptions about low back pain and its management: a gap between expectations and reality?. Health Expect.

[21] Goubert L., Crombez G., De Bourdeaudhuij I. (2004). Low back pain, disability and back pain myths in a community sample: prevalence and interrelationships. Eur J Pain.

[22] Ihlebæk C., Eriksen H.R., Ihlebaek C. (2004). The "myths" of low back pain: status quo in Norwegian general practitioners and physiotherapists. Spine.

[23] Vujcic I., Stojilovic N., Sipetic-Grujicic S. (2018). Low back pain among medical students in Belgrade (Serbia): a cross-sectional study. Pain Res Manage.

[24] Patel T., Chang F., Mohammed H.T. (2016). Knowledge, perceptions and attitudes toward chronic pain and its management: a cross-sectional survey of frontline pharmacists in Ontario, Canada. PLoS One.

[25] Munigangaiah S., Basavaraju N., Jadaan D.Y. (2016). Do "Myths" of low back pain exist among Irish population? A cross-sectional study. Eur J Orthop Surg Traumatol.

[26] Igwesi-Chidobe C.N., Coker B., Onwasigwe C.N. (2017). Biopsychosocial factors associated with chronic low back pain disability in rural Nigeria: a population-based cross-sectional study. BMJ Global Health.

[27] Walker B.F., Muller R., Grant W.D. (2004). Low back pain in Australian adults. Health provider utilization and care seeking. J Manipulat Physiol Ther.

[28] Pierobon A., Policastro P.O., Solino S. (2020). Beliefs and attitudes about low back pain in Argentina: a cross-sectional survey using social media. Musculoskel Sci Pract.

[29] Vargas-Prada S., Serra C., Martínez J.M. (2013). Psychological and culturally-influenced risk factors for the incidence and persistence of low back pain and associated disability in Spanish workers: findings from the CUPID study. Occup Environ Med.

[30] Sadeghian F., Coggon D., Ntani G. (2015). Predictors of low back pain in a longitudinal study of Iranian nurses and office workers. Work.

[31] Foster N.E., Bishop A., Thomas E. (2008). Illness perceptions of low back pain patients in primary care: what are they, do they change and are they associated with outcome?. Pain.

[32] Glattacker M., Heyduck K., Meffert C. (2012). Illness beliefs, treatment beliefs and information needs as starting points for patient information-evaluation of an intervention for patients with chronic back pain. Patient Educ Couns.

[33] Byrns G., Agnew J., Curbow B. (2002). Attributions, stress, and work-related low back pain. Appl Occup Environ Hyg.

[34] Byrns G., Reeder G., Jin G. (2004). Risk factors for work-related low back pain in registered nurses, and potential obstacles in using mechanical lifting devices. J Occup Environ Hyg.

[35] Ostelo R.W., Stomp-van den Berg S.G., Vlaeyen J.W. (2003). Health care provider's attitudes and beliefs towards chronic low back pain: the development of a questionnaire. Man Ther.

[36] Hayden J.A., Wilson M.N., Riley R.D. (2019). Individual recovery expectations and prognosis of outcomes in non-specific low back pain: prognostic factor review. Cochrane Database Syst Rev.

[37] PdC Silva Parreira, CG Maher, Latimer J. (2015). Can patients identify what triggers their back pain? Secondary analysis of a case-crossover study. Pain.

[38] Linton S.J., Warg L.E. (1993). Attributions (beliefs) and job satisfaction associated with back pain in an industrial setting. Percept Mot Skills.

[39] Lindström I., Ohlund C., Nachemson A. (1994). Validity of patient reporting and predictive value of industrial physical work demands. Spine.

[40] Christe G., Darlow B., Pichonnaz C. (2021). Changes in physiotherapy students' beliefs and attitudes about low back pain through pre-registration training. Arch Physiother.

[41] Zusman M. (1984). Spinal pain patients' beliefs about pain and physiotherapy. Aust J Physiother.

[42] Talbott N.R., Bhattacharya A., Davis K.G. (2009). School backpacks: it's more than just a weight problem. Work.

[43] Dean S., Hudson S., Hay-Smith E. (2011). Rural workers' experience of low back pain: exploring why they continue to work. J Occup Rehabil.

[44] Maeda A., Tsuji H., Naruse Y. (1997). Risk indicators of low back pain among workers in Japan: association of familial and physical factors with low back pain. Spine.

[45] Ree E., Lie S.A., Eriksen H.R. (2016). Reduction in sick leave by a workplace educational low back pain intervention: a cluster randomized controlled trial. Scand J Public Health.

[46] Keeley P., Todd C., Borglin G. (2008). Psychosocial predictors of health-related quality of life and health service utilisation in people with chronic low back pain. Pain.

[47] Scholey M., Hair M.D. (1989). The problem of back pain in physiotherapists. Physiother Pract.

[48] Adhikari S., Dhakal G. (2014). Prevalent causes of low back pain and its impact among nurses working in Sahid Gangalal National Heart Centre. J Nepal Health Res Counc.

[49] Battista S., Sansone L.G., Prevalence Testa M. (2021). Characteristics, association factors of and management strategies for low back pain among italian amateur cyclists: an observational cross-sectional study. Sports Med Open.

[50] French P., Flora L.F.W., Ping L.S. (1997). The prevalence and cause of occupational back pain in Hong Kong registered nurses. J Adv Nurs.

[51] Alshehri M.A., Alzahrani H., Alotaibi M. (2020). Physiotherapists' pain attitudes and beliefs towards chronic low back pain and their association with treatment selection: a cross-sectional study. BMJ Open.

[52] Ross M.D., Childs J.D., Middel C. (2014). Physical therapist vs. family practitioner knowledge of simple low back pain management in the U.S. Air Force. Mil Med.

[53] Werner E.L., Wormgoor M.E.A., Lindh E. (2007). Peer support in an occupational setting preventing LBP-related sick leave. Occup Med.

[54] Stevens M.L., Steffens D., Ferreira M.L. (2016). Patients' and physiotherapists' views on triggers for low back pain. Spine.

[55] Fitzgerald K., Vaughan B., Fleischmann M. (2020). Pain knowledge, attitudes and beliefs of Australian osteopaths drawn from a nationally representative sample of the profession. J Bodyw Mov Ther.

[56] Mehok L.E., Miller M.M., Hirsh A.T. (2019). Pain intensity and attribution mediate the impact of patient weight and gender on activity recommendations for chronic pain. J Pain Res.

[57] Benny E., Evans C. (2020). Ontario musculoskeletal physiotherapists' attitudes toward and beliefs about managing chronic low back pain. Physiother Can.

[58] Ihlebaek C., Eriksen H.R. (2005). Myths and perceptions of back pain in the Norwegian population, before and after the introduction of guidelines for acute back pain. Scand J Public Health.

[59] Adams S.R., Hacker M.R., McKinney J.L. (2013). Musculoskeletal pain in gynecologic surgeons. J Minim Invasive Gynecol.

[60] Boschman J.S., van der Molen H.F., Sluiter J.K. (2012). Musculoskeletal disorders among construction workers: a one-year follow-up study. BMC Musculoskelet Disord.

[61] James C., James D., Nie V. (2018). Musculoskeletal discomfort and use of computers in the university environment. Appl Ergon.

[62] Cherkin D.C., MacCornack F.A., Berg A.O. (1988). Managing low back pain - a comparison of the beliefs and behaviors of family physicians and chiropractors. West J Med.

[63] Brennan G., Shafat A., Mac Donncha C. (2007). Lower back pain in physically demanding college academic programs: a questionnaire based study. BMC Musculoskelet Disord.

[64] Werner E.L., Wormgoor M.E.A., Indahl A. (2008). Low back pain media campaign: no effect on sickness behaviour. Patient Educ Couns.

[65] Maselli F., Esculier J.F., Storari L. (2021). Low back pain among Italian runners: a cross-sectional survey. Phys Ther Sport.

[66] Tarimo N., Diener I. (2017). Knowledge, attitudes and beliefs on contributing factors among low back pain patients attending outpatient physiotherapy treatment in Malawi. South Afr J Physiother.

[67] Dabbous M.K., Moustafa S.M., Sakr F.R. (2020). Knowledge, attitude and practice of Lebanese community pharmacists with regard to self-management of low back pain. Trop J Pharmaceut Res.

[68] Ross M., Adams K., Engle K. (2018). The knowledge of low back pain management between physical therapists and family practice physicians. J Manual Manipulat Ther.

[69] Lobo M.E., Kanagaraj R., Jidesh V. (2013). An insight into adolescents' knowledge and attitudes on low back pain and its occurrence. Int J Ther Rehabil.

[70] Jolley D., Buchbinder R. (2007). Improvements in general practitioner beliefs and stated management of back pain persist 4.5 years after the cessation of a public health media campaign. Spine.

[71] Visuri T., Ulaska J., Pulkkinen P. (2001). Impact of chronic low back pain on military service. Mil Med.

[72] Li Y., Scholten-Peeters G.G.M., Coppieters M.W. (2020). How do people in China think about causes of their back pain? A predominantly qualitative cross-sectional survey. BMC Musculoskelet Disord.

[73] Roussel N.A., Neels H., Kuppens K. (2016). History taking by physiotherapists with low back pain patients: are illness perceptions addressed properly?. Disabil Rehabil.

[74] Werner E.L., Gross D.P., Lie S.A. (2008). Healthcare provider back pain beliefs unaffected by a media campaign. Scand J Prim Health Care.

[75] Houben R.M.A., Ostelo R.W.J.G., Vlaeyen J.W.S. (2005). Health care providers' orientations towards common low back pain predict perceived harmfulness of physical activities and recommendations regarding return to normal activity. Eur J Pain.

[76] Wolters P.M.J.C., Ostelo R.W.J.G., Stomp-van den Berg S.G.M. (2003). Health care provider's attitudes and beliefs towards chronic low back pain: the development of a questionnaire. Man Ther.

[77] Lefevre-Colau M.-M., Rannou F., Mace Y. (2009). Frequency and interrelations of risk factors for chronic low back pain in a primary care setting. PLoS One.

[78] Osborne A., Finnegan G., Blake C. (2013). An evaluation of low back pain among farmers in Ireland. Occup Med.

[79] Igumbor E.U., Useh U., Madzivire D.M. (2003). An epidemiological study of work-related low back pain among physiotherapists in Zimbabwe. South Afr J Physiother.

[80] Abdel Shaheed C., Mak W., Maher C.G. (2015). The effects of educational interventions on pharmacists' knowledge, attitudes and beliefs towards low back pain. Int J Clin Pharm.

[81] Abdel Shaheed C., Graves J., Maher C (2017). The effects of a brief educational intervention on medical students' knowledge, attitudes and beliefs towards low back pain. Scand J Pain.

[82] Johnsen T.L., Eriksen H.R., Baste V. (2019). Effect of reassuring information about musculoskeletal and mental health complaints at the workplace: a cluster randomized trial of the at work intervention. J Occup Rehabil.

[83] Odeen M., Ihlebæk C., Indahl A. (2013). Effect of peer-based low back pain information and reassurance at the workplace on sick leave: a cluster randomized trial. J Occup Rehabil.

[84] Staples M., Jolley D., Buchbinder R. (2009). Doctors with a special interest in back pain have poorer knowledge about how to treat back pain. Spine.

[85] McCabe E., Jadaan D., Munigangaiah S. (2019). Do medical students believe the back pain myths? A cross-sectional study. BMC Med Educ.

[86] Kaptein A.A., Van Ittersum M.W., Van Wilgen C.P. (2013). Do illness perceptions of people with chronic low back pain differ from people without chronic low back pain?. Physiotherapy.

[87] Coggon D., Ntani G., Palmer K.T. (2012). The cupid (cultural and psychosocial influences on disability) study: methods of data collection and characteristics of study sample. PLoS One.

[88] Darlow B., Perry M., Stanley J. (2014). Cross-sectional survey of attitudes and beliefs about back pain in New Zealand. BMJ Open.

[89] Steffens D., Maher C.G., Ferreira M.L. (2014). Clinicians' views on factors that trigger a sudden onset of low back pain. Eur Spine J.

[90] Kent P., Keating J.L., Kent P. (2005). Classification in nonspecific low back pain: what methods do primary care clinicians currently use?. Spine.

[91] Wolter T., Szabo E., Becker R. (2011). Chronic low back pain: course of disease from the patient's perspective. Int Orthop.

[92] Campbell C., Muncer S.J. (2005). The causes of low back pain: a network analysis. Soc Sci Med.

[93] Pagare V.K., Dhanraj T., Thakkar D. (2015). Beliefs about low back pain: status quo in Indian general population. J Back Musculoskel Rehabil.

[94] Pincus T., Santos R., Foster N.E. (2007). Attitudes to back pain amongst musculoskeletal practitioners: a comparison of professional groups and practice settings using the ABS-mp. Man Ther.

[95] Leysen M., Nijs J., Van Wilgen P. (2021). Attitudes and beliefs on low back pain in physical therapy education: a cross-sectional study. Braz J Phys Ther.

[96] Bar-Zaccay A., Bailey D. (2018). The attitudes and beliefs of UK osteopaths towards the management of low back pain: a cross-sectional study. Int J Osteopath Med.

[97] Ihlebæk C., Eriksen H.R. (2003). Are the 'myths' of low back pain alive in the general Norwegian population?. Scand J Public Health.

[98] Grimshaw J.M., Eccles M.P., Steen N. (2011). Applying psychological theories to evidence-based clinical practice: identifying factors predictive of lumbar spine x-ray for low back pain in UK primary care practice. Implement Sci.

