# Supplementary material for: What do people believe to be the cause of low back pain? A scoping review
Source: Braz J Phys Ther. 2023 Nov 7;27(6):100562. doi: 10.1016/j.bjpt.2023.100562 (PMC10679815; doi:10.1016/j.bjpt.2023.100562)
Supplement: Supplementary file 1 — Supplemental Material Supplemental material A: Search strategy Supplemental material B: Consensus chart for causal belief items Supplemental material C: Full list of items forming the categories of beliefs [file mmc1.pdf]

Supplementary material A

Medline Search Strategy

1 dorsalgia.mp.
2 back pain.mp.
3 backache.mp.
4 (lumb$ adj3 pain).mp. [mp=title, abstract, original title, name of substance word, subject heading word, floating sub-heading word, keyword heading word, organism supplementary concept word, protocol supplementary concept word, rare disease supplementary concept word, unique identifier, synonyms]
5 coccyx.mp.
6 coccydynia.mp.
7 sciatica/
8 ischialgia.mp.
9 spondylosis.mp.
10 lumbago.mp.
11 back disorder$.mp.
12 spinal pain.mp.
13 back trouble.mp.
14 1 or 2 or 3 or 4 or 5 or 6 or 7 or 8 or 9 or 10 or 11 or 12 or 13
15 ((cause* or causal* or pain or health or illness or disease or etiology or aetiology) adj3 (belie* or perception* or perceive* or attitude* or cognition or misconception*)).mp.
16 exp attitude to health/
17 14 and (15 or 16)

Basic Search | Find Citation | Search Tools | Search Fields | **Advanced Search** | Multi-Field Search

1 Resource selected | Hide | Change

Ovid MEDLINE(R) ALL 1946 to January 07, 2022

Enter keyword or phrase (* or $ for truncation) ◉ Keyword ○ Author ○ Title ○ Journal

Search | Expand Term Finder

▸ Limits (expand) ☐ Include Multimedia ☑ Map Term to Subject Heading

▼ Search History (17) View Saved

#	Searches	Results	Type	Actions	Annotations
17	14 and (15 or 16)	4515	Advanced	Display Results | More	
16	exp attitude to health/	449838	Advanced	Display Results | More	
15	((Cause* or causal* or pain or health or illness or disease or etiology or aetiology) adj3 (Belie* or perception* or perceive* or attitude* or cognition or misconception*)).mp.	365311	Advanced	Display Results | More	
14	1 or 2 or 3 or 4 or 5 or 6 or 7 or 8 or 9 or 10 or 11 or 12 or 13	83103	Advanced	Display Results | More	
13	back trouble.mp.	111	Advanced	Display Results | More	
12	spinal pain.mp.	1656	Advanced	Display Results | More	
11	back disorder$.mp.	657	Advanced	Display Results | More	
10	lumbago.mp.	1444	Advanced	Display Results | More	
9	spondylosis.mp.	5279	Advanced	Display Results | More	
8	ischialgia.mp.	137	Advanced	Display Results | More	
7	Sciatica/	5113	Advanced	Display Results | More	
6	coccydynia.mp.	173	Advanced	Display Results | More	
5	coccyx.mp.	1628	Advanced	Display Results | More	
4	(lumb$ adj3 pain).mp. [mp=title, abstract, original title, name of substance word, subject heading word, floating sub-heading word, keyword heading word, organism supplementary concept word, protocol supplementary concept word, rare disease supplementary concept word, unique identifier, synonyms]	5621	Advanced	Display Results | More	
3	backache.mp.	3783	Advanced	Display Results | More	
2	back pain.mp.	67983	Advanced	Display Results | More	
1	dorsalgia.mp.	114	Advanced	Display Results | More	

Save | Remove | Combine with: AND | OR

Save All | Edit | Create RSS | Create Auto-Alert | View Saved

Email All Search History | Copy Search History Link | Copy Search History Details

Supplementary Material B

Consensus chart for causal belief items

Items included as investigating a causal belief marked in green.

**Pain Attitudes and Belief Scale for Physiotherapists (PABS-PT)** (1, 2)	Subscale orientation	Causal belief
Mental stress can cause back pain even in the absence of tissue damage	Biopsychosocial	yes
The cause of back pain is unknown	Biopsychosocial	yes
Pain is a nociceptive stimulus, indicating tissue damage	Biomedical	yes
A patient suffering from severe pain will benefit from physical exercise	Biopsychosocial	No
Functional limitations associated with back pain are the result of psychosocial factors	Biopsychosocial	no
Patients with back pain should preferably practice only pain free movements	Biomedical	No
Therapy may have been successful even if pain remains	Biopsychosocial	no
Back pain indicates the presence of organic injury	Biomedical	yes
If back pain increases in severity, I immediately adjust the intensity of treatment accordingly	Biomedical	no
If therapy does not result in a reduction in back pain, there is a high risk of severe restrictions in the long term	Biomedical	no
Pain reduction is a precondition for the restoration of normal functioning	Biomedical	no
Increased pain indicates new tissue damage or the spread of existing damage	Biomedical	yes
There is no effective treatment to eliminate back pain	Biopsychosocial	no
Even if the pain has worsened, the intensity of the next treatment can be increased	Biopsychosocial	no
If patients complain of pain during exercise, I worry that damage is being caused	Biomedical	yes
The severity of tissue damage determines the level of pain	Biomedical	yes
Learning to cope with stress promotes recovery from back pain	Biopsychosocial	no
Exercises that may be back straining should not be avoided during the treatment	Biopsychosocial	no
In the long run, patients with back pain have a higher risk of developing spinal impairments	Biomedical	no

**Back Pain Attitudes Questionnaire (Back-PAQ)** (3) (4)..	Theme	Causal belief
You can injure your back and only become aware of the injury sometime later	Vuln	no
Lifting without bending the knees is not safe for your back	Vuln	No
If you overuse your back, it will wear out	Vuln	No
Sitting is bad for your back	Vuln	No
It is easy to injure your back	Vuln	no
Bending your back is good for it	Vuln	No
Your back is one of the strongest parts of your body	Vuln	No
A twinge in your back can be the first sign of a serious injury	Vuln	yes
Your back is well designed for the way you use it in daily life	Vuln	no

Good posture is important to protect your back	Prot	no
It is important to have strong muscles to support your back	Prot	no
You could injure your back if you are not careful	Prot	no
If your back hurts, you should take it easy until the pain goes away	Prot	no
If an activity or movement causes back pain, you should avoid it in the future	Prot	no
If you ignore back pain, you may cause damage to your back	Pain	no
Expecting your back pain to get better helps you to recover from back pain	Pain	no
Focusing on things other than your back helps you to recover from back pain	Pain	no
Worrying about your back can delay recovery from back pain	Pain	no
When you have back pain, you can do things which increase your pain without harming the back	Pain	no
Back pain means that you have injured your back	Pain	yes
Stress in your life (financial, work, relationship) can make back pain worse	Pain	no
Thoughts and feelings can influence the intensity of back pain	Pain	no
There is a high chance that an episode of back pain will not resolve	Prog	no
Most back pain settles quickly, at least enough to get on with normal activities	Prog	no
Once you have had back pain there is always a weakness	Prog	no
Once you have a back problem, there is not a lot you can do about it	Prog	no
Having back pain makes it difficult to enjoy life	Sp	no
It is hard to understand what back pain is like if you have never had it yourself	Sp	no
To effectively treat back pain you need to know exactly what is wrong	Sp	no
It is important to see a health professional when you have back pain	Sp	no
It is worse to have pain in your back than your arms or legs	Sp	no
When you have back pain the risks of vigorous exercise outweigh the benefits	Acti	no
If you have back pain you should avoid exercise	Acti	no
If you have back pain you should try to stay active	Acti	no
Vuln: vulnerability of the back (questions are about your own back)<br>Prot: The need to protect the back (looking after your own back)<br>Pain: the relationship between pain and injury<br>Sp the special nature of back pain (back pain in general)<br>Acti: activity participation while experiencing pain (what you should do if you have back pain)<br>Prog: prognosis of back pain (recovering from back pain)		

**Pain Belief Questionnaire (PQB)** (5).	Subscale orientation	Causal belief
Pain is the result of damage to the tissue of the body.	Organic	yes
Physical exercise makes pain worse.	Organic	no
It is impossible to do much for oneself to relieve pain.	Organic	no
Being anxious makes pain worse.	Psychological	no

Experiencing pain is a sign that something is wrong with the body.	Organic	yes
When relaxed pain is easier to cope with.	Psychological	no
Being in pain prevents you from enjoying hobbies and social activities.	Organic	no
The amount of pain is related to the amount of damage.	Organic	yes
Thinking about pain makes it worse.	Psychological	no
It is impossible to control pain on your own.	Organic	no
Pain is a sign of illness.	Organic	yes
Feeling depressed makes pain seem worse.	Psychological	no

**Attitudes to back pain scale in musculoskeletal practitioners (ABS-MP)** (6).	Section	Domain	Causal belief
I explore the psychological problems that my patient is facing.	PI	PS	no
It is essential that I know about my patients' psychological difficulties.	PI	PS	no
I often find myself providing psychological support to patients.	PI	PS	no
I try to avoid probing into my patients' personal problems. (R)	PI	PS	no
I am concerned about the quality of treatment my referred patients receive.	PI	CC	no
I don't believe that there is anyone out there who could help my back pain patients more than I do. (R)	PI	CC	no
Regular treatment by a physical therapist does not help prevent back pain. (R)	PI	LS	no
I believe in continuing to treat the patient after the back pain has been resolved, to prevent its return.	PI	LS	no
If I keep seeing patients on and off I can prevent relapse.	PI	LS	no
If I keep seeing patients on and off, they might never learn to manage their back problem themselves. (R)	PI	LS	no
When I refer my patients I know they will be seen within a suitable time frame.	PI	CHS	no
I don't see myself as connected to a health system of resources that I can access. (R)	PI	CHS	no
When referring patients I am confident they will receive good treatment.	PI	CHS	no
If you look hard enough you can find a structural reason for most patients' back pain.	TO	BM	yes
I advise back pain patients to restrict their life-style.	TO	BM	no
I often find I have to teach patients to be vigilant about their backs.	TO	BM	no
The most important goal of treatment is to increase mobility.	TO	RA	no
Return to normal daily activities is the most important consequence of treatment.	TO	RA	no

My objective is to get my patients back to work quickly.	TO	RA	no
PI: Personal interaction attitudes, TO: Treatment orientation, PS: Psychologic, CC: Confidence and concern, LS: limitations on sessions,<br>CHS: Connection to health care system, BM: biomedical beliefs, RA: Return to work and daily activity increasing mobility			

**Survey of pain attitudes (SOPA) Harm Subscale** (7)	Causal belief
The pain I usually experience is a signal that damage is being done	yes
Pain is a signal that I have not been exercising enough	yes
Pain does not necessarily mean that my body is being harmed	yes
Exercise and movement are good for my pain problem	no
If I exercise, I could make my pain problem much worse	no
Something is wrong with my body which prevents much movement or exercise	no
If I do not exercise regularly, my pain problem will continue to get worse	no
Exercise can decrease the amount of pain I experience	no

**Survey of pain attitudes (SOPA) Emotional Subscale** (7)	Causal belief
Nothing but my pain really bothers me	no
Anxiety increases the pain I feel	no
My pain is not emotional, it is purely physical	yes
Stress in my life increases my pain	no
Depression increases the pain I feel	no
My pain is mostly emotional, and not so much a physical problem	yes
There is a strong connection between my emotions and my pain level	no
No matter how I feel emotionally, my pain stays the same	no

**Neurophysiology pain test (NPQ)**(8)	Causal belief
Receptors on nerves work by opening ion channels in the wall of the nerve.	no
When part of your body is injured, special pain receptors convey the pain message to your brain.	no
Pain only occurs when you are injured or at risk of being injured.	yes
Special nerves in your spinal cord convey ‘‘danger’’ messages to your brain	no
Pain is not possible when there are no nerve messages coming from the painful body part.	yes
Pain occurs whenever you are injured	yes
The brain sends messages down your spinal cord that can change the message going up your spinal cord.	no
The brain decides when you will experience pain.	no
Nerves adapt by increasing their resting level of excitement.	no
Chronic pain means that an injury hasn’t healed properly	yes
The body tells the brain when it is in pain.	no
Nerves can adapt by producing more receptors.	no
Worse injuries always result in worse pain.	yes
Nerves adapt by making ion channels stay open longer.	no
Descending neurons are always inhibitory.	no

When you injure yourself, the environment that you are in will not affect the amount of pain you experience, as long as the injury is exactly the same	no
It is possible to have pain and not know about it.	no
When you are injured, special receptors convey the danger message to your spinal cord	no
All other things being equal, an identical finger injury will probably hurt the left little finger more than the right little finger in a violinist but not a piano player.	no

**Fear avoidance belief questionnaire (FABQ)**(9)	Causal belief
My pain was caused by physical activity	yes
Physical activity makes my pain worse	no
Physical activity might harm my back	no
I should not do physical activities which (might) make my pain worse	no
I cannot do physical activities which (might) make my pain worse	no
My pain was caused by my work or by an accident at work	yes
My work aggravated my pain	no
I have a claim for compensation for my pain	no
My work is too heavy for me	no
My work makes or would make my pain worse	no
My work might harm my back	no
I should not do my normal work with my present pain	no
I cannot do my normal work with my present pain	no
I cannot do my normal work till my pain is treated	no
I do not think that I will be back to my normal work within 3 months	no
I do not think that I will ever be able to go back to that work	no

**Tampa scale of kinesiophobia (TSK)**(10)	Causal belief
I’m afraid that I might injury myself if I exercise	no
If I were to try to overcome it, my pain would increase	no
My body is telling me I have something dangerously wrong	yes
My pain would probably be relieved if I were to exercise	no
People aren’t taking my medical condition seriously enough	no
My accident has put my body at risk for the rest of my life	no
Pain always means I have injured my body	yes
Just because something aggravates my pain does not mean it is dangerous	no
I am afraid that I might injure myself accidentally	no
Simply being careful that I do not make any unnecessary movements is the safest thing I can do to prevent my pain from worsening	no
I wouldn’t have this much pain if there weren’t something potentially dangerous going on in my body	yes
Although my condition is painful, I would be better off if I were physically active	no
Pain lets me know when to stop exercising so that I don’t injure myself	no
It’s really not safe for a person with a condition like mine to be physically active	no
I can’t do all the things normal people do because it’s too easy for me to get injured	no
Even though something is causing me a lot of pain, I don’t think it’s actually dangerous*	no

No one should have to exercise when he/she is in pain	no
*We are not sure whether dangerous refers to the pain or the "something" causing the pain	

**Myths and other statements**	Causal belief
Most backpain is caused by injury of heavy lifting	Yes
There is nothing physically wrong with many patients with LBP	yes
Most often it will be possible to find an exact cause of the pain	yes
Modern X-ray will usually find the cause of the back pain	yes
Is the cause of your LBP known?	yes
this pain or discomfort could be due to prolonged sitting and working on digital screens in lockdown	yes
X-ray and newer imaging tests can always identify the cause of pain	yes

References:

1. Ostelo RW, Stomp-van den Berg SG, Vlaeyen JW, Wolters PM, de Vet HC. Health care provider's attitudes and beliefs towards chronic low back pain: the development of a questionnaire. Man Ther. 2003;8(4):214-22.
2. Houben RM, Ostelo RW, Vlaeyen JW, Wolters PM, Peters M, Stomp-van den Berg SG. Health care providers' orientations towards common low back pain predict perceived harmfulness of physical activities and recommendations regarding return to normal activity. European journal of pain (London, England). 2005;9(2):173-83.
3. Darlow B, Perry M, Mathieson F, Stanley J, Melloh M, Marsh R, et al. The development and exploratory analysis of the Back Pain Attitudes Questionnaire (Back-PAQ). BMJ open. 2014;4(5):e005251.
4. Christe G, Nzamba J, Desarzens L, Leuba A, Darlow B, Pichonnaz C. Physiotherapists' attitudes and beliefs about low back pain influence their clinical decisions and advice. Musculoskeletal science & practice. 2021;53:102382.
5. Edwards LC, Pearce SA, Turner-Stokes L, Jones A. The Pain Beliefs Questionnaire: an investigation of beliefs in the causes and consequences of pain. Pain. 1992;51(3):267-72.
6. Pincus T, Vogel S, Santos R, Breen A, Foster N, Underwood M. The attitudes to back pain scale in musculoskeletal practitioners (ABS-mp): the development and testing of a new questionnaire. The Clinical journal of pain. 2006;22(4):378-86.
7. Jensen MP, Turner JA, Romano JM, Lawler BK. Relationship of pain-specific beliefs to chronic pain adjustment. Pain. 1994;57(3):301-9.
8. Moseley L. Unraveling the barriers to reconceptualization of the problem in chronic pain: the actual and perceived ability of patients and health professionals to understand the neurophysiology. The journal of pain. 2003;4(4):184-9.
9. Waddell G, Newton M, Henderson I, Somerville D, Main CJ. A Fear-Avoidance Beliefs Questionnaire (FABQ) and the role of fear-avoidance beliefs in chronic low back pain and disability. Pain. 1993;52(2):157-68.
10. Bunzli S, Smith A, Watkins R, Schütze R, O'Sullivan P. What Do People Who Score Highly on the Tampa Scale of Kinesiophobia Really Believe?: A Mixed Methods Investigation in People With Chronic Nonspecific Low Back Pain. Clin J Pain. 2015;31(7):621-32.

Supplementary material C

Full list of items forming the categories of beliefs

	CATEGORIES														
	**Lifting and bending**	**Physical Activity and sports**	**Loading, movements and physical capacity**	**Physical work demands**	**Other work demands**	**Posture or position**	**Structural injury or impairment**	**Trauma mechanism**	**Genetic**	**Mental or psychological**	**General health and lifestyle**	**External environment**	**Spirutal**	**Unknown**	**Other**
ITEMS AS PRESENTED IN THE INCLUDED STUDIES	Carrying	Walking	Unexpected loads	Vibrations	Lack of proper work organization	Posture	Structural	Sport injuries	Genetic	Stress	Poor health status	Social factors	Five elements imbalance	The cause of back pain is unknown.	Previous LBP episodes
	Lifting	Physical activities	Sudden movement	Continuing to work when injured or hurt	Work scheduling	Fixed posture	Sciatica	Crash	Heredity	Financial pressure	Diabetes	Environmental	Energy status	I don't know the cause of my LBP	Risk factor
	Object not close to body	Physical exercise	Unanticipated sudden movement or falls by patients	Crutching/shearing	Improper work techniques	Awkward posture	Arthritis	Accident at home	Natural/gender	Psychological trauma	Menstruation and kidney status	Environmental pollution	Spiritual	Unknown cause	Risk taking for fast results
	Do you have any pain at the present time that you believe is due to wearing your backpack/bag?	Garden work	Coughing/sneezing	Farm work	Inadequate training in injury prevention.	Poor posture	Joint problems	Work-related injury	Genetic disorder	Emotional problems	Painful/heavy menstruation	Familial problems	External fate	Do not know	Previous history of LBP and physically demanding work
	Not bending knees	House cleaning	Distracted	Physical work demand	Poor working environment	Postural	Muscle disorder	Trauma		Emotional status	Diet/nutrition	Not wearing good shoes	External - fate/luck	Accident/chance	Behavioural factors
	Lifting and bending	Work I did at home	Unstable/unbalanced/difficult to grasp or hold	Nurse actions	Heavy mental workload	Bad position	Spine/pelvis/lower limb impairment	Accident/trauma		Psychological	Unhealthy diet	Bad mattress		Chance or bad luck	Poor self-care<br><br>Internal attributions

	Bending	Cycling	Bending or twisting your back in an awkward way	Once-off specific injury related to using marchinery/tools/implements	Working conditions	Half-rising position	Weak muscles	Physical trauma		Mental condition	Alcohol	Sleeping in poor bed		Coincidence/bad luck	Personality
	Frequent bending	Gardening	Unaccustomed activity	Once-off specific injury related to animal handling	Working with confused or agitated patients	Maintaining specific posture at work	Back spasm	Trauma and injuries to the back		My mental attidude	Smoking	Live people/animals			Individual
	Bending and twisting	Moderate or vigorous physical activity	Physical exertion	Monotonous work	Professionel workload	Awkward posture	Diminished trunk muscle strength and fatigability	Traumatic		Lack of interest management	Hormonal changes	Draughts			Physical factors
	Twisting	Sexual activity	Lack fitness for task	Assisting patient during gait activities	Overworked	Lying down	Most often, it will be possible to find an exact cause of the pain	Once-off specific injury related to other		Psychological factors	Blood flow	Heat or cold			Caring for a sick relative
	Heavy loads	ATV/quad bike use	Not enough rest breaks during the day	Reaching or working away from your body	Workload	Driving	Biomechanical	Accident or injury		Psychological/psychosocial	Sleeping disorders	Weather conditions			Fatigue
	Injury or heavy lifting	Horse riding	Vigorous physical activity only	Performing manual orthopedic techniques	Rapid work pace	Driving or being in a car	Postural problems, joint problems and slipped disc	Accident		Mental stress can cause back pain even in the absence of tissue damage	Overweight				Fatigue /tired
	Bending and twisting while lifting	Running	Repeated movements	Treating a large number of patients in one day	LBP is commonly caused by people's work	Standing	LBP as biomedical problem	As the result of an injury		Psychosomatic	Having a serious disease				My own behavior
	Poor posture while lifting heavy objects	Strength and fitness training	Other repeated actitvity	Working in the same position for long periods	My LBP was caused	Prolonged standing	Spine/pelvis/lower limb impairment	Falling		Mental stress during an exam period	Having caught a cold				Other

					by my work										
	Lifting and twisting	Regular exercise is not a cause for LBP	Repeated lifting/pushing/pulling	Working in awkward or cramped positions	Beliefs that professional activities were responsible for LBP	Standing for too long	Spinal disease	Once-off specific injury related to trips/slips/falls		Stress/worries	Menopause				
	Once-off specific injury related to lifting/pushing/pulling	Sport	Unilateral physical stress is not a cause of back pain	Accident at work	Poor working conditions	Sitting	There is nothing physically wrong with many patients with chronic back pain	Slip/trip/fall		Psychological stress	Due to hysterectomy				
	Poor lifting of heavy loads	Sport activity	Overuse	Physical workplace	External job tasks	Prolonged sitting	Bacterial or viral	Accident at work			Illness				
	Lifting and carying	Team sport	Performing the same task over and over	Chronic heavy work/wear and tear	Low back pain problems are commonly caused by people's work	Sitting at the university	Changes in neural system	Occupational accident			As a consequence of a related disease				
	Lifting leading to injury leading to low back pain (LBP)	Individual sport	Squatting	Sedentary work	Working conditions cause back injuries to U.S. nurses	Poor sitting	Back pain means that you have injured your back				Medical				
	Heavy lifts	Contact within sports		Do not use safety assistance devices	Caused by work	Sitting for too long	The severity of tissue damage determines				Surgery				

							the level of pain.								
	Frequent bending and twisting	Intensive sport activities		Heavy physical workload	The type of job done at work	Improper body posture	Increased pain indicates new tissue damage or the spread of existing damage.				No painkillers				
	Bending or twisting your back in an awkward way	Physical inactivity		Lifting or transferring dependent patients		Bad posture	Pain is a nociceptive stimulus, indicating tissue damage.				Bad medical care				
		Lack of exercise		Carrying, lifting or moving heavy materials or equipment		Sitting in the same posture for a long period of time	If patients reported of pain during exercise, I worry that damage is being caused.				Side-effect of other medication				
		Physical activity and sport (too much/too less)		Lifting heavy load/patients		stooping	Back pain indicates the presence of organic injury.				Pregnancy Getting older/aging				
		Unbalanced life style /too much or too little exercise)		Lifting patient within bed with assistance			A twinge in your back can be the first sign of a serious injury				LBP can affect lean people				
		Physical inactivity		Lifting patient within bed without assistance			Pain only occurs when you are injured or at risk of being injured				Immunity				

				Repeated tractor driving			Pain is not possible when there are no nerve messages coming from the painful body part				Immunit y change s				
				Pulling/pushing			Pain occurs whenever you are injured				Back pain is not usually due to any serious disease				
				Repeated animal handling			Chronic pain means that an injury hasn't healed properly								
				Performing surgery Work-related factors			Worse injuries always result in worse pain								
				Working at or near your physical limit			CLBP is always related to injury								
				Leaning over patient			LBP is always related to injury								
				Restraining struggling patient			pain was thought to be due to either a muscle, disc or joint out of place								
				Giving assistance when patient starts to fall			pain was thought to be due to torn								

							muscles or ligaments								
				Ambulating patient			People with backache often have a slipped disc or trapped nerve.								
				Transferring patient (bed to chair; bed to bed)			Modern X-rays will usually find the cause of the back pain								
				Lifting patient from floor without assistance			If you look hard enough you can find a structural reason for most patients' back pain								
				Lifting patient from floor with assistance			Radiographs and newer imaging tests (computed tomography [CT] and magnetic resonance imaging [MRI] scans) can always identify the cause of pain								
							X-ray and imaging tests (scans) can always identify the cause of back pain								

							muscle strain								
							vertebral subluxation								
							facet joint syndrome								
							disc problem								
							spinal arthritis								
							X-rays or a CT scan will determine the cause of low back pain								
							Leg length discrepancy								
							facet								
							contained disc								
							instability								
							sacroiliac								
							trapped nerves								
							Other biomechanical risk factors								
